# A tandem giant magnetoresistance assay for one-shot quantification of clinically relevant concentrations of N-terminal pro-B-type natriuretic peptide in human blood

**DOI:** 10.1007/s00216-021-03227-5

**Published:** 2021-02-23

**Authors:** Fanda Meng, Weisong Huo, Jie Lian, Lei Zhang, Xizeng Shi, Aldo Jesorka, Yunhua Gao

**Affiliations:** 1 Institute of Basic Medicine, The First Affiliated Hospital of Shandong First Medical University, Jinan, 250014 China; 2 Institute of Basic Medicine, Shandong First Medical University & Shandong Academy of Medical Sciences, Jinan, 250062 China; 3 Department of Chemistry and Chemical Engineering, Chalmers University of Technology, SE-412 96 Gothenburg, Sweden; 4 Dongguan Bosh Biotechnologies, Ltd., Dongguan, 523808 China; 5 College of Criminal Investigation, People’s Public Security University of China, Beijing, 100038 China; 6 Key Laboratory of Photochemical Conversion and Optoelectronic Materials, Technical Institute of Physics and Chemistry, Chinese Academy of Sciences, Beijing, 100190 China; 7 University of Chinese Academy of Sciences, Beijing, 100149 China

**Keywords:** POCT, Microfluidic, GMR, Biosensor, NT-proBNP

## Abstract

We report a microfluidic sandwich immunoassay constructed around a dual-giant magnetoresistance (GMR) sensor array to quantify the heart failure biomarker NT-proBNP in human plasma at the clinically relevant concentration levels between 15 pg/mL and 40 ng/mL. The broad dynamic range was achieved by differential coating of two identical GMR sensors operated in tandem, and combining two standard curves. The detection limit was determined as 5 pg/mL. The assay, involving 53 plasma samples from patients with different cardiovascular diseases, was validated against the Roche Cobas e411 analyzer. The salient features of this system are its wide concentration range, low detection limit, small sample volume requirement (50 μL), and the need for a short measurement time of 15 min, making it a prospective candidate for practical use in point of care analysis.

## Introduction

Heart failure (HF) is a common, costly, and potentially deadly condition. In developed countries, around 2% of the adult population suffers from heart failure. In those over the age of 65, this percentage increases to 6–10% [1]. By means of clinical data, a strong association between the N-terminal pro-B-type natriuretic peptide (NT-proBNP) level and the mortality in patients with heart failure has been established [2]. NT-proBNP is considered the gold standard biomarker in heart failure [3–5].

Currently, detection of NT-proBNP is still confined to the medical laboratory. The analysis is based on conventional immunoassay tests, whose results may require several hours or even days to be delivered [6]. Rapid and easily performed NT-proBNP quantification in human blood could reduce the time requirement, and would allow for real-time monitoring, even by the patient. Such personalized diagnostics option may avoid unnecessary or improper treatment and hospital admission, which for the benefit of the patient aids in preventing permanent heart damage, and ultimately results in reduced medical expenses and preserved resources.

Point-of-care testing (POCT) is a rapid and simple means for diagnosing diseases which could be done by health care workers without technical training [7]. Superior to traditional laboratory detection, POCT can be performed at bedside by non-laboratory-trained personnel and increase efficiency to clinical decision-making about additional testing or guiding therapy [8]. Time of transport and preparation of clinical samples can be reduced, and test results are rapidly available at the point of care. For example, cardiac diseases can be diagnosed as soon as the symptoms appear; therefore, the mortality and morbidity can be effectively decreased [9, 10].

With the advent of microfluidics technology, it has become established that microfluidics is particularly suitable for POCT, due to its intrinsic merits of low reagent and sample consumption, low cost, and the possibility of integration with miniaturized detectors [11–14]. Microfluidics has, for example, been combined with various immunosensing elements for glucose, various ions, proteins, and DNA. In 1992, Abbot Point of Care Inc. introduced the i-Stat system, utilizing microfluidics technology to quantify various inorganic cations and glucose [15]. The i-Stat testing cartridge was subsequently expanded to allow for the quantitative measurement of cardiac troponin I in whole blood or plasma [16]. Similarly, the Triage cardiac panel by Biosite Inc. is based on fluorescence immunosensing in a microfluidic chip format, and can detect three cardiac biomarkers simultaneously [17].

Giant magnetoresistance (GMR) is the change in resistance of some materials in response to an applied magnetic field. GMR technology was initially exclusively used to read data in hard disk drives, but has since become widespread in biomedical sensing applications. For example, Schotter et al. had shown that the GMR sensor was more sensitive than comparable standard fluorescent DNA detection [18]. Zhi et al. had developed a novel HBV genotype detecting system based on the GMR sensor, and magnetic nanoparticles (MNPs) [19]. Gaster et al. adopted GMR sensors for quantification of protein interactions [20]. GMR sensing technology combines high sensitivity with portability, low cost, and real-time detection. In addition, the fabrication of GMR sensors is compatible with conventional cleanroom technologies devised for mass production, so the cost of GMR sensors can be greatly reduced, enabling the development and fabrication of POCT devices.

**Fig. 1:**
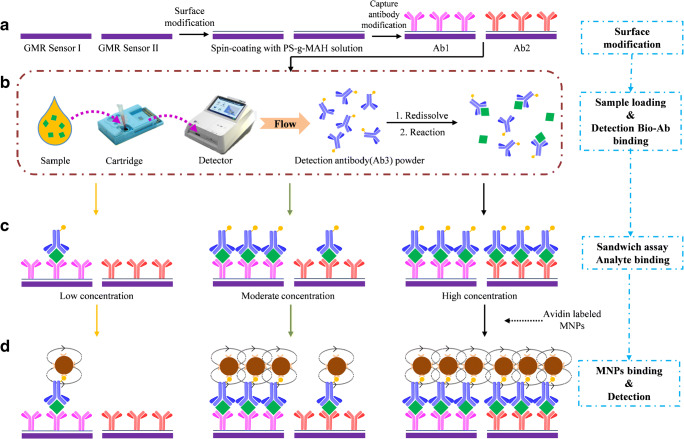
The assay protocol. **a** Surface modification. Sensor I and II represent the different GMR sensors, and Ab1 and Ab2 represent the respective capture antibodies. **b** Sample loading and detection antibody binding. Yellow dots represent the biotin tags for binding to the avidin-coated magnetic nanoparticles. **c** Analyte binding. **d** Magnetic particle binding and detection

In this paper, we present a dual-sensor POCT chip device, combining sandwich immunoassays with a microfluidic sample handling device. The two integrated GMR sensors utilized in tandem in the assay system are individually coated with detection antibodies of different affinity to the antigen, leading to differential binding at different concentration ranges, which extends the dynamic range of the tandem detector, compared to the individually operated devices (Fig. 1). On the other hand, compared with the currently commercial kit, the developed POCT chip device can be performed more easily at bedside by non-laboratory-trained personnel, which just needed sample injecting and chip inserting. The integrated microfluidic assay reduced the sample volume requirement and was very suitable for multi-detection.

In order to widen the measurement range to cover the concentrations relevant for HF diagnosis, we used two kinds of capture antibodies (Fig. 1a). In the presence of low concentrations of antigen, capture antibody Ab1, deposited on sensor I, generates a response on GMR sensor I. With increasing concentration of antigen, GMR sensor I becomes eventually saturated, while the response of sensor II increases. In the intermediate situation with moderate analyte concentration, we combined the two signals by means of averaging the standard curves obtained from the two sensors at their respective overlapping concentration ranges.

The clinically relevant concentration range for NT-proBNP, determined with commercial assays (e.g., Roche Diagnostics Elecsys® proBNP Immunoassay), is between 5 pg/mL and 35,000 pg/mL. The recommended clinical threshold has been suggested as 125 pg/mL for patients younger than 75 years of age, and 450 pg/mL for patients 75 years and older. For the latter case, NT-proBNP < 300 pg/mL is indicative of the absence of acute HF [21].

## Materials and methods

### Reagents

All reagents used in this work were of analytical grade. Potassium dihydrogen phosphate (KH_2_PO_4_), sodium dihydrogen phosphate (NaH_2_PO_4_), potassium chloride (KCl), and sodium chloride (NaCl) were obtained from Alfa Aesar. Tween 20 was obtained from AMRESCO (USA). For the detection of NT-proBNP antigens, two anti-NT-proBNP monoclonal antibodies (Ab1, 15C4, and Ab2, 11D1) were combined as capture antibody, and one anti-NT-proBNP monoclonal antibody (Ab3, 13G12) as detection antibody. All antibodies as well as recombinant NT-proBNP were purchased from HyTest, Ltd. (Finland). Clinical human plasma samples were provided by the Peking University Shenzhen Hospital. The detection antibody was biotinylated using NHS-biotin (Alfa Aesar) [22].

Avidin-coated MNPs used as magnetic tags were purchased from Ademtech, Ltd. (France). A sample of polystyrene-grafted-maleic anhydride (PS-g-MA, graft ratio 17%) was provided as a free sample by Longjia Plastics Fabrication (Jilin, China).

**Fig. 2:**
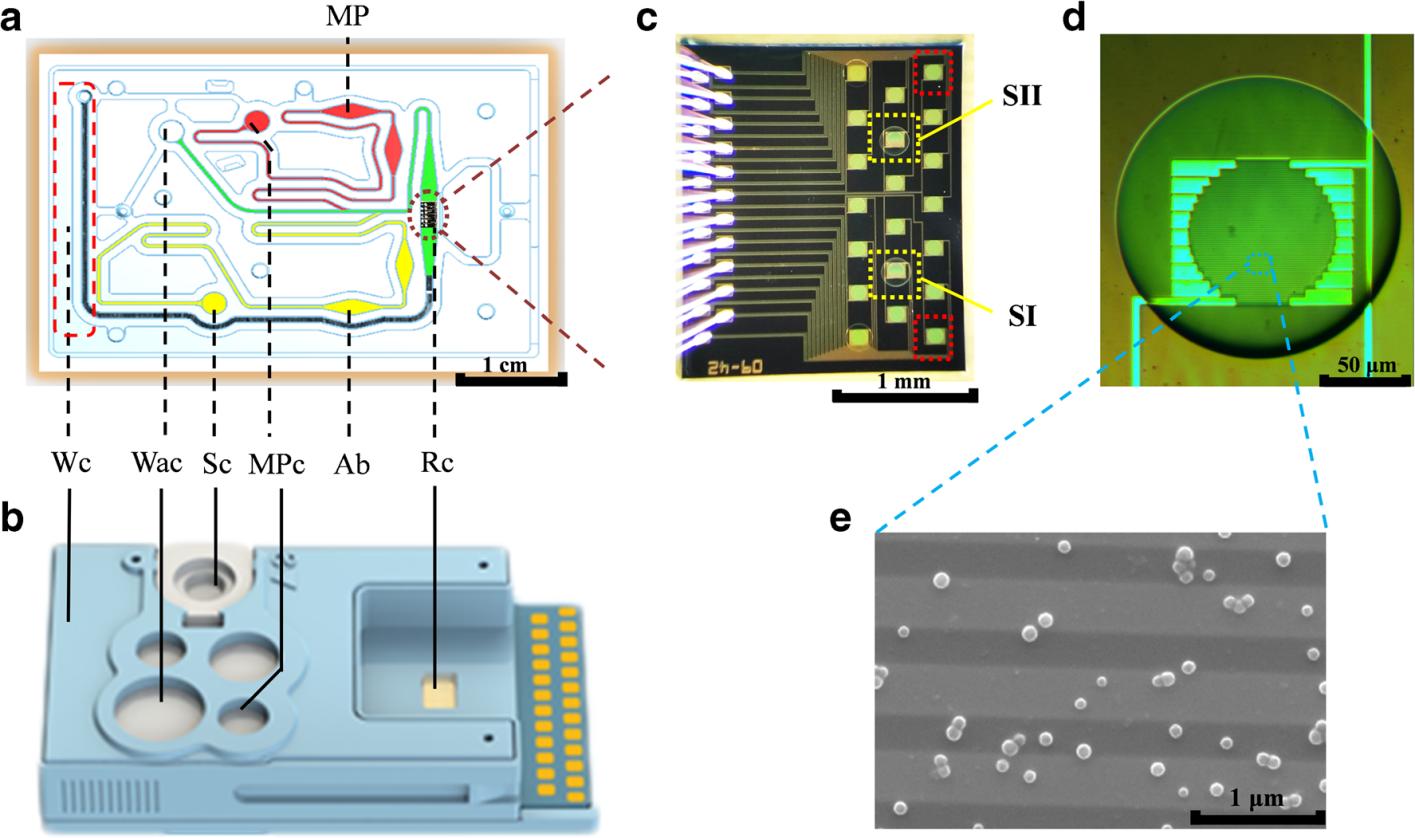
The assay system used in the experiments. **a** Schematic drawing of the chip layout [23]. The deposition areas for avidin-coated magnetic particles (MP) and biotinylated detection antibodies (Ab) are marked. Wc, waste cavity; Wac, washing buffer cavity; Sc, sample cavity; MPc, magnetic nanoparticle rehydration buffer cavity; Rc, reaction cavity. **b** POCT cartridge, consisting of the microfluidic sample handling circuitry with on-chip cavities, the sensor array chip, and a contact pad interface. **c** GMR sensor array. The two selected sensor units SI and SII used in tandem are marked in yellow. Two uncoated sensors (TC) used for calculating a compensating factor to account for temperature changes during the measurement are marked in red. **d** Magnification of a single sensor unit, covered with a droplet of capture antibody during incubation. The border between droplet and sensor surface (silicon wafer chip) gives under the microscope the impression of a shallow cavity. **e** SEM micrograph of the sensor surface after MNP (*gray spheres*) binding

The assay cartridge (Fig. 2) was developed by Dongguan Bosh Biotechnologies, Ltd. (China), and is part of a GMR-based assay processor. Figure 2b shows the main components of the assay cartridge. Details have been published elsewhere [23]. The cartridge consists of a 20-unit GMR sensor array on a 2 mm × 2.3 mm chip, integrated into a microfluidic sample handling unit with on-chip wells.

### Device preparation

The GMR sensor used in this study is a multilayer thin-film structure on a Si wafer (Si (450 μm)/SiO_2_ (10 nm)) composed of Ta (4.5 nm)/PtMn (10 nm)/CoFe (2 nm)/Cu (2 nm)/CoFe (1 nm)/NiFe (3 nm)/Al_2_O_3_ (40 nm). The chip (Fig. 2c) has a size of 2.0 mm × 2.3 mm, and contains 20 individual GMR sensor units with a diameter of 90 μm each.

The sensor surface (Fig. 2d) was modified by spin coating with PS-g-MA toluene solution (1% w/v) [24], using a spin coater (KW-4A, Institute of Microelectronics of the Chinese Academy of Sciences, I: 800 rpm, 30 s, II: 2000 rpm, 60 s). The specimen was subsequently dried at 60 °C in thermal convection oven (ZD-85, Jintan Jincheng Guosheng Experimental Instrument Factory, China) for 10 min. The two capture antibody solutions (50 μg/mL in carbonate buffer, 0.1 M, pH = 9.6) were printed onto the specified positions of the sensor array surface by means of a Nano-Plotter NP 2.1 (GeSiM, Germany) and incubated at 37 °C and 70% humidity for 30 min.

Prior to assembly of the assay cartridge (Fig. 2b), the detection antibody and the MNP were pipetted onto designated areas of the channel layer (Fig. 2a), and freeze-dried. This allowed for storage of the prepared device for up to 6 months.

### Assay procedure

The surfaces of the sensor units were pre-coated with the recognition antibodies (Fig. 1a. Nanoparticles and detection antibodies were also pre-deposited on the cartridge (cf. Fig. 2b), to be re-hydrated during the assay. The sandwich assay protocol consists in the first step of an immunoreaction of antigen (sample or standard), introduced through designated on-chip wells, with detection antibodies. Simultaneously, the antigen binds to the capture antibodies on the surface, forming the assay sandwich (5 min at RT, Fig. 1b, c). Subsequently, the binding to avidin-coated magnetic nanoparticles (dissolved in carbonate buffer, 0.1 M, pH = 9.6) is initiated (5 min at RT, Fig. 1d). Intermittent washing steps (10 mM PBS, pH = 7.4, 0.5% Tween 20) are applied in between the binding reactions. The captured MNPs (Fig. 2d) are finally detected by the GMR sensors (Fig. 2e), the outputs of which are processed in order to obtain the calibration curves and sample measurements. The entire assay process is completed within 15 min.

### Data collection and processing

For the construction of the calibration curves, three reference measurements were performed per data point, and means and standard deviations were calculated. A total of 58 patient samples were analyzed; one measurement was performed per patient sample. We obtained two standard curves for the two individual sensor responses, reflecting the difference in binding affinity, and thus antigen concentration. The standard curve A covers the concentration range from 15 to 5000 pg/mL, and standard curve B covers the range from 100 to 40,000 pg/mL. The values for the middle range from 100 to 5000 pg/mL were determined by calculating the mean of the individual sensor-specific concentrations obtained from standard curves A and B.

## Results

### Optimization of detection performance

**Fig. 3:**
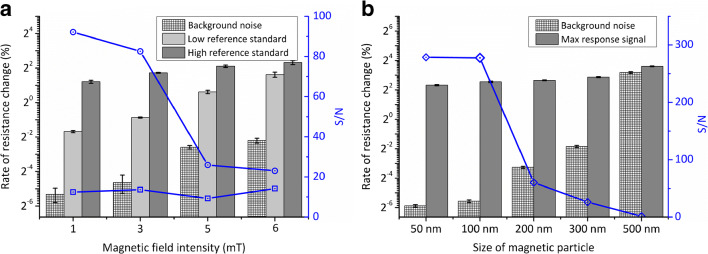
Optimization of the detection (*n* = 3). **a** The dependence of detector signal (*bars*) and signal to noise ratio (S/N, lines) on the magnetic field intensity. Hollow squares: S/N of the low concentration reference standard; hollow circles: S/N of the high concentration reference standard. **b** The influence of the size of magnetic particle on the detector response (*bars*), and the S/N ratio (*line*)

We investigated the effects of magnetic field intensity and the size of the MNPs on the measurement performance. The influence of the magnetic field strength on the immune reaction was observed in the presence of 0 (blank), 100 (low concentration reference standard), and 5000 (high concentration reference standard) pg/mL NT-proBNP. With increasing magnetic field strength, the background noise, as determined from the blank measurement, and the detection signal increase accordingly (Fig. 3a). The S/N ratio for the low concentration reference standard has its maximum value at a field strength of 3 mT (30 G). A field of 3 mT shows acceptable performance for both low and high concentration standards of NT-proBNP. Consequently, a 3-mT field was used in the experiments.

We investigated the relationship between the maximum response signal and the size of magnetic particles used in the individual assays. We found that the magnitude of the maximum response is increasing with particle size, and more than doubles over the range between 50 and 500 nm (Fig. 3b). However, with particles of Ø 200 nm and larger, the background increased such that the S/N decreases sharply. It is likely that larger particles have the tendency to remain in the detection area due to non-specific adsorption to the sensor surface. We conclude that the optimal size of the MNPs is 100 nm for this assay, and have chosen this size for all experiments.

### Calibration of the NT-proBNP assay

**Fig. 4:**
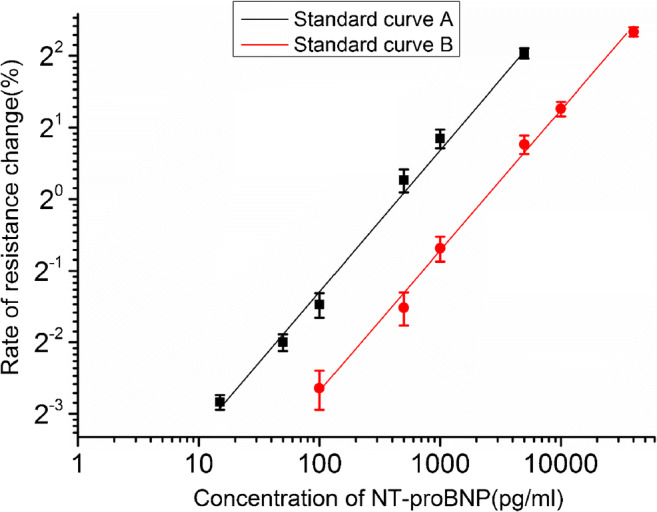
The standard curves (*n* = 3) obtained with a low concentration reference standard on GMR sensor I (*black line*) and a high concentration reference standard on GMR sensor II (*red line*). Each data point is the average of three repeat measurements, each carried out using a single assay cartridge. The error bars indicate ± 1 σ

Using the optimal system parameters, the relationships between rate of resistance change and concentration of NT-proBNP (Fig. 4) was determined for both sensors. Standard curve A is the plot of the calibration function of the antigen concentration (linear fit with log c as variable) from 15 to 5000 pg/mL (Y = 0.0263X^0.595^, *r* = 0.994), standard curve B is the same for sensor B and 100 to 40,000 pg/mL (Y = 0.0103X^0.590^, *r* = 0.998). The limit of detection (3S/N) was found to be 5 pg/mL.

### Validation

**Fig. 5:**
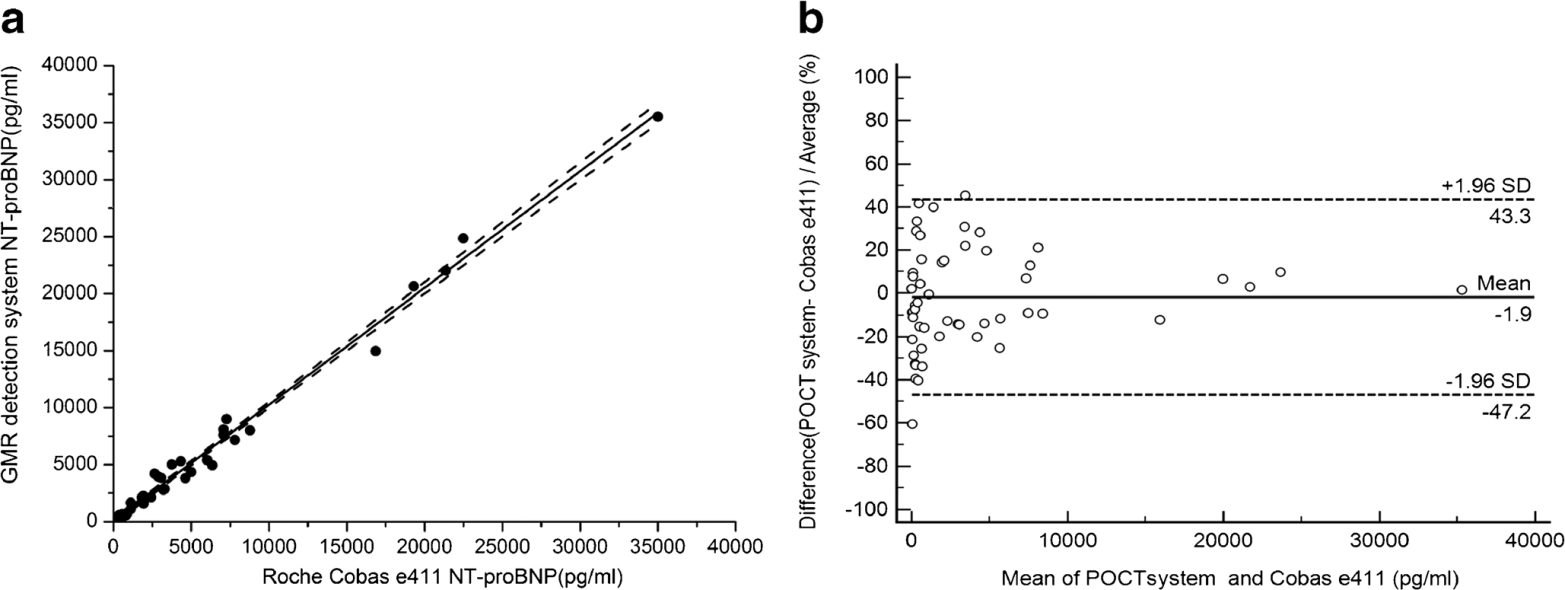
Method comparison between two assays. Passing-Bablok regression analysis (**a**) and Bland-Altman analysis of agreement between the GMR and the Roche Cobas e411 assay (**b**)

The assay results obtained with the GMR detection system were validated against the Roche Cobas e411 assay (Fig. 5). Passing-Bablok regression analysis yielded the following equation for the data set covering the entire concentration range (*n* = 53): y = 1.02x + 10.68; *r* = 0.995 (Fig. 5a). The 95% confidence interval for the slope is 1.00 to 1.05, and for the y intercept, the 95% confidence interval is − 70.06 to 91.42. Figure 5b shows the Bland-Altman plot of the relative differences between the data sets of both assays. The mean relative difference (95% confidence limit) is 1.9% (− 47.2 to 43.3%). There is no statistically significant bias between the two assays.

## Discussion

The tandem operation of two GMR sensors with different dynamic ranges in a microfluidic sample handling cartridge expands the dynamic range to cover the full clinically relevant concentration range of NT-proBNP. In the commercial assay analyzer, the sensors in the array are used in parallel for repeat measurements, but not in tandem.

In order to apply differential antibody coating to the GMR sensors, a facile surface modification method based upon the functional polymer PS-g-MAH was introduced, using a combination of spin coating and nano-plotting. A 1% solution of the polymer was used in the procedure, since earlier studies revealed that at that concentration, the film quality was such that the highest degree of surface immobilization of antibody could be achieved, while the film quality was highest. The roughness of the film increased with greater polymer concentration. It is conceivable that the optimal concentration can vary for different antibodies, but this was not investigated further. A slight drawback for further application development is the currently limited commercial availability of research amounts of the grafting polymer, although the material itself is produced on a large scale for plastics consumer goods. One cartridge is required per assay; re-use strategies should be investigated for improved sustainability.

The approach is open for surface functionalization with different antibodies, making the assay concept easily expandable to a wide variety of analytes.

Data treatment by means of two combined standard curves increased the detection range for a single assay by one order of magnitude. Validation against a commercial assay revealed that the accuracy of the method would be acceptable in a clinical testing setting. The assay’s lightweight instrumental back end, short measurement time of ~ 15 min, and simple operation are beneficial pre-requisites for point of care application. The required sample size is only 50 μL.

Although some essential figures of merit of the new tandem method are comparable to the established commercial assay processor as well as to the Roche analyzer used in the validation study, e.g., LOD and LOQ (Table 1); others were clearly improved.

**Table 1:** Comparison of GMR and Roche Cobas e411 assay

	GMR assay	^1^Roche assay
LOD (pg/mL)	5	5
LOQ (pg/mL)	15	50
Assay time (min)	15	18
Sample volume (μL)	50	15
Measuring range (pg/mL)	5–40,000	5–35,000

^1^Specification from Roche, Ltd.

The most important aspect is the abovementioned range expansion. This is coupled to a reduced time and lower sample requirement for a full range analysis: both are influential performance parameters in order to achieve a high degree of portability and simplicity expected from POC instrumentation.

## Concluding remarks

We have presented a promising sample handling and detection system for wide-range quantitative NT-proBNP determination in human plasma, with favorable figures of merit as well as distinct operational benefits. The tandem operation of several GMR units on the same chip is a technical framework for functionally combining identical sensing units. One alternative to be explored is the use of different assay formats on the same lab on a chip, which can be most likely implemented without changes to the chip or sensor design. For example, by a combination of a competitive and a sandwich assay, the hook effect can be directly detected in order to effectively cover the full diagnostic concentration range of a biomarker.
